# Some Points in the Pathology of Tubercle

**Published:** 1880-09

**Authors:** J. M. Da Costa


### Selections.

---

Some Points in the Pathology of Tubercle. By J. M. Da Costa, m.d. Read at the meeting of the Pathological Society of Philadelphia, April 22, 1880.

In attempting to put together some thoughts on the pathology of tubercle, it will be necessary, however briefly, to refer to the unsettled state of the question in the best medical minds of the day. Immediately following Laennec, nothing could have appeared more firmly fixed than the doctrine he so clearly enunciated. It was impossible to doubt tuberculosis as a specific disease. To have misgivings as to the nature of consumption and its constant association with tubercular destruction was to appear to return to barbaric darkness. Not to separate with clearness the different forms of tubercle was to forfeit all claim to be a pathologist. But we all know what has recently happened. The German iconoclast has been at work. Nobody likes to speak now of tubercular diathesis, of tubercle being a constitutional affection. It is for the most part simply the result of a local inflammation; and cheesy matter, infective process from absorption, irritation in structures abounding in lymphatic tissues, are the complacent phrases of the day, which satisfy most as much now as diathesis, constitutional condition, specific deposit, satisfied most not many years since.

And the local view, if such it may be called, once adopted, has brought with it scores of interesting observations on the inoculation of tubercle; its artificial causation; its production in the lung by inhalation of both tubercular and non-tubercular substances—observations which are warmly discussed, criticised, adopted, rejected, explained, explained away, and the uncertainties connected with which, quite apart from the other difficulties of the subject, are the cause mainly of the generally disturbed condition of the whole inquiry.

Underlying these observations, or at least closely connected with them, lies the vital question, What relation does tubercle bear to the inflammation? And it is this question particularly that I desire to examine with you a little more fully to-night, and concerning which I shall venture to offer the result of some researches and reflections.

As a necessary introduction, I shall have to examine the evidence on which we pronounce a mass to be tubercular; in other words, what its minute structure as shown by the microscope is. And, to avoid any confusion at the threshold of our inquiry, let me speak of that which we find in undoubted tubercle, in the little, hard, miliary bodies, which may afterwards become aggregated into larger, gray masses. In them we encounter three elements:

*Medium-sized, rather shriveled cells*, not very regular in outline, consisting of finely-granular, dense material, with a nucleus small in proportion to the cells, or with several nuclei of similar character. They were once regarded as significant of tubercle, but they are now supposed to be swollen epithelial cells which have undergone retrograde metamorphosis. Mixed up with them are cells less dense and like ordinary epithelium, small cells like leucocytes, and a great deal of granular material of doubtful origin.

*Giant cells.* These consist of large, many-nucleated cells, which are found at rather an advanced stage of tuberculosis, and are very marked in the acute form. They are of spheroidal shape, and somewhat irregular outline. Great stress has been laid on them as significant of tubercle, but they have been met with in deposits in various tissues of the body, in scrofula, in syphilis, and in merely hyperplastic lymph-glands of those perfectly free from tubercle.* As they grow they send out long, branched processes. With Klein, I believe them to be excessively developed or fused epithelial cells.

---

* Weiss, Virchow's Archiv, lxviii.

The structure in which all these cellular elements are found, especially, perhaps, those last described, is *a fine net-work* like the fine trabecular net-work in the interior of lymphatic glands; and this led to the belief entertained until recently by Rindfleisch, that tubercle is a lymphoid growth. But this is not stating the whole of the manner and arrangement of the cells in tubercle. They are found in the lungs filling the alveoli and infiltrating—generally as small, round cells—the alveolar walls, and leading to very considerable thickening of the latter.

To sum up, cell-growths by themselves, not peculiar, but representing different grades of development—some still rapidly growing, others shrivelling and full of dense matter; all capable of being washed out of a fine reticulum, or accumulating in masses both within and in the walls of air-vesicles—this structure, this grouping, may be regarded as tubercle. Then there are certain secondary alterations that take place in the tubercular formation and the invaded tissue which must also be mentioned, and which bespeak a retrograde change and low vitality. The main of these changes is a degeneration of the cell-growths, an accumulation of granules and fatty material, and an occlusion of the pulmonary capillaries, probably from pressure, and here and there a fibroid transformation of the giant cells.

Now, what causes all this? Some still maintain a specific non-inflammatory deposit; some say an inflammatory process of slight intensity; others a specific inflammation. I pick up a recent journal, and see that malaria is at the bottom of all this strange cell-growth and rapid decay. I turn to one of this month, and I find in the front of the periodical an article proving that tubercle has its origin in disorders in the trophic centers, and in the middle pages another, showing that it is an accident, the result of the capillary interference, due to altered condition of the blood from the presence of yeast. It is almost needless to say that the bacteria are made to explain the peculiar formations, for how could these patient little beings that are bearing so quietly being made the scapegoats of the pathologists of the last half of the nineteenth century, escape having charged to them this additional sin? I turn with eagerness to discussions of the subject replete with learning in societies similar to ours, and there is little but negation. It is not this, and not that, say men who are known wherever medicine is cultivated; and you begin to doubt if there is such a thing as tubercle at all, until the first clinic-room you go into—and see the familiar face, hear the cough, and recognize the well-known signs—confronts you with the stern reality of the awful disease. With all these doubts and gropings after the truth, I may be pardoned if I hold fast to the belief that the process, whatever it be, is something special, though something of which we do not hold the key.

To return from this digression to one part of the subject around which much of what is positive in our knowledge has clustered, and which is of most obvious applicability—the relation of these mysterious tubercular formations to inflammation.

Now, we all know how the relation of tubercle to inflammation has engaged the attention of the present generation of pathologists. Yet the consideration of the question long antedated them; and the much-neglected observations of that sagacious thinker, Addison, are really the key-note to many of the views now brought forward under other names. But this is a historical issue, with which we cannot further concern ourselves here. The active discussion of the matter started with the observation of Virchow that the caseous matter previously regarded as infiltrated tubercle might originate from the fatty degeneration of diverse morbid products, and was non-tubercular; indeed, that the gray granulations alone were tubercular and non-inflammatory. Niemeyer expanded this thought, and engrafting on it the doctrine of Buhl, of the infection of tubercle, promulgated the view that the lung consolidation and destruction were most commonly inflammatory—the result of the caseous pneumonia—and the tubercle, when met with, quite secondary and accidental. Indeed, to carry out Niemeyer's ideas logically, the inflammation is, in the vast majority of instances, everything, the tubercle nothing.

The doctrine of Buhl has been alluded to, that tuberculosis—as seen, for instance, in its most typical form of miliary tubercle—is due to infection from masses of cheesy material. The infection happens chiefly through the lymphatics. This infection theory led to numerous experiments on animals, with the result that inoculation in rabbits and guinea-pigs with fresh miliary tubercle, with cheesy matter, with the sputa from tubercular patients, has been followed by acute miliary tuberculosis. Moreever, going still further, Cohnheim and Fraenkel have shown that it is unnecessary to inoculate with these special matters, for in rabbits and guinea-pigs the formation of any focus of suppurative inflammation may fill the viscera with tubercles. With reference to these experiments, it has been pointed out that the kind of animal on which they are made has much to do with the the result. Rabbits and guinea-pigs are particularly prone to tuberculosis. Yet, as regards inoculation with tubercular matter, it has also succeeded on other animals, as in the experiments of Böllinger on goats.*

Another group of experiments must be alluded to,—those in which pulverized tubercle and cheesy matter have been forced into the lung by inhalation. These have been followed by tubercular-looking nodules; and so have, as Schottelius† has recently demonstrated, inhalations with non-tubercular substances, such as pulverized calf's brain and cheese, produced apparently identical bodies. They are the results of inflammatory irritation. But, microscopically examined, they do not present the appearances of tubercle.

Summing up all the experimental observations, they seem to me to prove that tubercle may be transmitted by inoculation either of true tubercular matter or so-called caseous pneumonia; that resorption of tubercle and infection of previously healthy parts is a view sustained by evidence; that the production of tubercle from non-tubercular material, either by inoculation or by inhalation, is not proved. Inflammatory nodules arise, but they have not the structure of tubercular formations.

I shall now attempt to answer the question what is the exact nature of those inflammatory changes in the lung which give rise to destructive consolidation, supposed to be non-tubercular, yet from which, by infection, tubercle may come. In other words, I shall endeavor to describe the histology of so-called "caseous pneumonia," or pneumonic phthisis. We find within

* Mittheilungen aus dem Pathologischen Institute zu München, 1878.

† Virchow's Archiv, lxxiii, 1878.

the alveoli an accumulation of large cellular elements, mixed with leucocytes and exudation matter. We observe the alveoli walls thickened and infiltrated with cells, the vessels compressed, accounting for the breaking down of the bloodless masses accumulated in and around the alveoli. We meet with inflammatory infiltration in the walls of the bronchi, and as Rindfleisch has so well pointed out, around them, as well as with increased connective tissue between the lobules and around the finer bronchial tubes. In studying the cellular masses we encounter the so-called giant cells. There is, indeed, nothing we do not find in this caseous pneumonia that we have not spoken of in tubercle, only the proportions are different and the admixture of the elements of inflammation more marked. These changes spread usually over a large portion of the lung, and, especially as regards the amount of connective tissue, are modified by the duration of the disease. One of the most striking of the lesions, and one which I have rarely failed to encounter in the many specimens examined, is the infiltration with small cells of the walls of the alveoli. Green,* too, regards them as very important, and Wilson Fox† looks upon them even as tubercular. As the cheesy matter degenerates, evidences of broken-down tissue, with considerable granular detritus, meet the eye. Yet Cohnheim‡ has recently told us that the cheesy part contains in reality but little fat.

Now, is there anything in all this which broadly separates this so-called caseous pneumonia in its minute structure from tubercle? Is there anything more than the evident admixture of a marked inflammatory lesion? Is there anything in the low vitality of the mass and its tendency to decay and fall asunder which is different? And if we call this affection at once "tubercular pneumonia," we are, I verily believe, much nearer to the truth than in endeavoring to separate it from tubercle altogether.

But it is not simply on histological grounds that I arrive at this conclusion. I have long studied the subject clinically, and can record it here as my deliberate opinion that the number of

---

* Pathology of Pulmonary Consumption, 1878.

† Transactions of Pathological Society of London, 1873.

‡ Die Tuberkulose vom Standpunkte der Infektionslehre, 1879.

cases of consumption which are supposed to have inflammatory beginnings is grossly exagerated. They are the exception, not the rule; and even in the cases in which we have had evidence of an active bronchitis or pneumonic condition having seemingly been the start of all the difficulty, how often do we not find, on close analysis, that failing health, hacking cough, even slight spitting of blood, have preceeded the acute symptoms? Then, too, we may get the history of inherited scrofulous or tuberculous diathesis. But I do not wish to be misunderstood. There are cases in which none of these qualifying elements can be discerned, which have, to all appearance, started in an acute inflammatory process. It is only the relative frequency of these cases that I am denying.

Again, I ask, what becomes of the instances of so-called pneumonic phthisis? Do they become tubercular? How many autopsies can any one recall, where persons dying from some intercurrent affection, while laboring under so-called pneumonic phthisis, did not show at some portion of the lung, miliary tubercle or larger masses which everybody would pronounce undoubted tubercle?

Now, admitting the connection of so-called "caseous pneumonia" or "pneumonic phthisis" with the subsequent development of tubercle,—and nobody denies this, whatever his views as to the character of the connection,—I believe that it is quite as logical to reason from the after-appearance of the tubercle as to the primary character of the so-called inflammation, as to reason from the inflammation and absorption of its products to the formation of the tubercle. The reasoning backward is as good as the reasoning forward, and, I think, infinitely more likely to be true.

Again, how many cases of ordinary pneumonia happening in perfectly healthy persons are met with which pass, no matter how, into tubercle? Certainly not many. When it occurs there is generally the history of scrofula or tubercle in the family, the taint. Many of the advocates of the inflammatory origin of tubercle, or of its subsequent development after inflammation, tacitly admit this when they speak of the inflammation as special or specific. If it is special or specific, I say at once it is tubercular,—tubercular either from the onset, or it has become so when it presents the appearance of caseous changes.

I am advocating, then, the view that caseous pneumonia leads to tubercle elsewhere, because it is really tubercle already ; and that it is not the products of ordinary inflammation, but the tubercular products, which infect. They may appear with the inflammation or be the result of a special kind of inflammation; that does not affect my argument.

Now, one great difficulty in admitting this argument is, that since the researches of Virchow have familiarized us with the facts we cannot assume all kinds of caseous degeneration as tubercular. We know that such changes may happen in purulent collections, in cancer, and that, microscopically, they present the features of the so-called cheesy degeneration which attends pneumonic phthisis. But is there nowhere else similarity of appearance without identity of meaning? Can we tell every case of cancer, under all circumstances, by its cell-growth alone? Are there no healthy textures in the course of formation that look like it? Does every sarcoma present infallible features at all its stages? Moreover, I have already stated that we very generally, nay, almost constantly, find in the pneumonic lesion much the same elements, mixed with the products of degeneration, which we recognize as tubercular in undoubted tubercle.

I believe, then, that pneumonic phthisis is a tubercular pneumonia, and that the inflammation is tubercular from the onset, or has acquired a tubercular nature by changes in the cell-life which we do not understand. Perhaps these are connected with sluggish tissue-change under the influence of a virus,—a taint inborn, or acquired by impure air and bad hygienic surroundings. That we cannot see these things in the protoplasm or cells with even our highest powers is no proof of their non-existence. Do we perceive the manly form of the athlete in the little spermatozoon? What do we find in the ovum to explain the transmission of the delicate features and matchless figure of one generation to the famed beauty of another? Where are, in either germs, the lurking tendencies to disease which we see constantly reproduced in families? Where the specks that indicate cancer, scrofula, tubercle, gout?

The tubercular inflammation may appear as such, and then we have a more or less acute character of case; or the tubercular action may not start for years afterwards. I shall show you some drawings taken from cases that I had watched for years.

Here is one from a woman of 45, who, under my observation, had for eight years a chronically-consolidated lung, non-tubercular. Sorrow overtook her, her general health failed; struggles with poverty came, and she became tubercular. You perceive here how the lower lobe of one lung had undergone the caseous change and contained tubercles, while the upper is simply dark with pigment and densely consolidated. A few scattered, comparatively recent miliary tubercles are found in the other lung.

Here is a yet more striking instance, where a man had for five years a lung which, you see, looks exactly like the red hepatization of ordinary pneumonia. Softening, without a vestige of tubercle, is occuring in parts. In a streak at the upper lobe tubercular pneumonia is evident; the other lung is entirely healthy.

Perhaps the views here advocated may appear to call in doubt the transmission of tubercle by resorption and infection,—its constant reproduction, as it were, and scattering through the system. But they do not. These observations are among the best sustained and most valuable in modern pathology, and they are all the easier to understand if we admit the starting-point of the infection to have been a tubercular inflammation. As a contribution to this doctrine of infection and absorption, and also as furnishing many points of analogy with the instances mentioned where inflammation of the lung has been followed by tubercular formation, let me show the remarkable specimens of abdominal tumors and tuberculosis on the table, taken from cases of mine that happened some years since at the Pennsylvania Hospital.

In the first case, occuring in a man the subject of syphilis, and much tormented with abdominal pains, a hard tumor was discovered in the right iliac fossa. This was followed, after some months, by marked emaciation, sweats, diffused abdominal tenderness, diarrhœa, and signs of deposit in the lungs. At the autopsy the mass you see here of dense fibrous tissue was found in the right iliac fossa, below the head of the colon; a small cavity containing pus was detected in its interior; on its exterior were tubercular nodules, The intestines, on their peritoneal surface, throughout their length were thickly studded with tubercular nodules; the mesenteric glands were enlarged and softened; there were miliary tubercles in the lungs.

In the second case, a man also broken down by syphilis, there was the same history of colics and cramps, a tumor was discovered in the right iliac fossa, three inches from the crest of the ilium. He had noticed the tumor for years. Gastric irritability, tenderness over the abdomen, ascites, diarrhœa, fever, cough, signs of lung-consolidation, became gradually prominent symptoms, and he died exhausted. A thick, firm mass of inflammatory matter was found covering the cæcum, and had occasioned the tumor. At one part, on the outer and lower wall of the cæcum, was a small cavity containing gelatinoid matter mixed with black, thin fluid. The ileum above the ileocæcal valve, as well as the inflammatory, hard tissue in this region, was covered with tubercles. The kidneys contained tubercles; in the left supra-renal capsules were several tubercular nodules. The lungs were full of small, gray, tubercular granulations.

Here, then, are two cases of strange similarity, in which a local inflammation in the abdomen was followed in time by diffused tubercle, both abdominal and pulmonary. But let us return to what I more particularly meant to discuss,—the formation of tubercle in the lungs, and its bearing on the different kinds of phthisis, especially pneumonic phthisis.

Will the views which I have endeavored to bring before you do away with the clinical distinctions concerning which we hear now so much? Not at all. If the so-called pneumonic phthisis has a separate history, gives points for special diagnosis and prognosis, requires different treatment, it ought, no matter what our views of its real pathology, to be recognized as a distinct form. But let us go no further; we are leaving the broad highway for rough and tortuous paths, when, as is the tendency of the present day, we attempt to sweep all cases of lung-destruction into the phthisical category.

What do we gain by reverting to a very old nomenclature, and by making phthisis a generic term? To carry this out, we ought even to abolish the word cancer, and speak of it as a cancerous phthisis, for surely there is also destruction of the lung enough in pulmonary cancer. The consequence of all this agitation about the word is nothing but confusion. Scarcely two persons seem to use phthisis in the same sense. There are forms of lung-destruction which are clearly non-tubercular; let us give them their special names, and not apply to them a term which has long been applied to that by far most common form of lung-destruction,—phthisis from tubercle. Pneumonic phthisis may be made a separate variety; but that ought to be all. Nay, I venture to suggest whether, in view of the idea long associated with the name of phthisis, and of what I still humbly believe is beyond comparison the most frequent cause of the consumption, it will not be better, unless some other form is particularized, still to retain the name for the tubercular malady, and to so understand it.

But I have claimed your indulgence long enough, and, in bringing these remarks to a close, I can only hope that some train of thought may have been suggested by them which your labors will brilliantly elucidate. *E scintilla lux;* from the flickering glimmer of to-night I trust through others for the steady glow on a subject that sadly needs light.

*—Philadelphia Medical Times.*

